# Favoring D_2_-Lymphadenectomy in Gastric Cancer

**DOI:** 10.3389/fsurg.2018.00042

**Published:** 2018-06-07

**Authors:** Ioannis Karavokyros, Adamantios Michalinos

**Affiliations:** First Department of Surgery, National and Kapodistrian University of Athens, Athens, Greece

**Keywords:** gastric cancer, lymphadenectomy, gastrectomy, D2-lymphadenectomy, D2-dissection, extended lymphadenectomy, radical lymphadenectomy

## Abstract

The role of extended lymphadenectomy in the surgical treatment of gastric cancer has been debated for many years. So far six prospective randomized trials and a number of meta-analyses comparing D_1_- to D_2_-lymphadenectomy in open surgery have been published with contradicting results. The possible oncologic benefit of radical lymphadenectomy has been blurred by a number of reasons. In most of the trials the strategies under comparison were made similar after protocol violations. Imperfect design of the trials could not exclude the influence of cofounding factors. Inappropriate endpoints could not detect evidently the difference between the two surgical strategies. On the other hand radical lymphadenectomy was characterized by increased morbidity and mortality. This was mostly caused by the addition of pancreatico-splenectomy in all D_2_-dissections, even when not indicated. A careful analysis of the available evidence indicates that D_2_-lymphadenectomy performed by adequately trained surgeons without resection of the pancreas and/or spleen, unless otherwise indicated, decreases Gastric Cancer Related Deaths and increases Disease Specific Survival. This evidence is not compelling but cannot be ignored. D_2_-lymphadendctomy is nowadays considered to be the standard of care for resectable gastric cancer.

## The Rationale for Extended Surgery

The benefit of resection of regional lymph nodes in gastric cancer been debated for many years and still remains under question. Τhe failure of limited surgery to control disease loco-regionally was reported as early as the 1950s by Gordon McNeer. In his autopsy series he found more than 20% cancer recurrence in the non-resected perigastric nodes or the gastric bed (1). As a remedy he proposed “a more thorough” operation with lymphadenectomy of the celiac axis and pancreatico-splenectomy (2). Although this operation improved loco-regional control, it didn’t affect survival (3) and didn’t gain popularity in the West.

In the East at that time the Japanese Research Society for Gastric Cancer established “General Rules” for the surgery of Gastric Cancer (4). The society classified the nodes participating in the lymphatic drainage of the stomach into tiers similarly to today’s classification. The perigastric nodes are the first tier, those lying along the big vessels are the second one, and the more remote, para-aortic lymph nodes nowadays constitute the third tier. Removal of the first tier constitutes D_1_ lymphadenectomy (formerly known as R_1_-resection), removal of the second tier D_2_ lymphadenectomy (formerly known as R_2_ resection), removal of the third a D_3_ lymphadenectomy. In the 1960’s the Japanese Society suggested that removal of the appropriate number of tiers would increase the chance of negative “lymphadenectomy margins”. Furthermore, under the influence of the work of Kanai, Kajitani and Aikou (5–7), pancreatosplenectomy became a standard part of D_2_-lymphadenectomy overlooking the fact that it required specific indications (8). Thus the typical Japanese operative strategy ended up pretty much similar to the one proposed by McNeer. Setting this operation as a standard the Japanese reported remarkably high survival rates. These were soon noticed by their Western colleagues and again the possible benefit of extended lymphadenectomy became a matter of investigation (9). Since then, a number of prospective randomized trials and meta-analyses have been conducted to compare D_2_- to D_1_-lymphadenectomy.

As discussed above, the concept of D_2_-lymphadenectomy was initially based on anatomical grounds. In agreement with the current 8^th^ edition of TNM the first and second lymph node tiers are considered local and regional respectively. An ideal gastrectomy with D_2_-lymphadenectomy corresponds to extirpation of all locoregional disease. However this “ideal” resection can be verified only by thorough examination of the operative field. Examination of the resected specimen cannot rule out the possibility of lymphatic tissue left behind. Alternatively, we can compare the number of resected lymph nodes to the number of lymph nodes that normally exist on each tier. Wagner et al demonstrated in a cadaveric study that D_2_-lymphadenectomy would lead to an average retrieval of 27 lymph nodes, but he also noted that significant differences exist among the individuals in the total number of lymph nodes (10). On the other hand, Sharma et al retrieved an average of 35 nodes with a D_2_-dissection again in a cadaveric study in India (11) . The variation between the two studies may reflect a racial difference between the two populations, or a more extensive dissection, or even the cofounding effect of “less hygienic food consumption leading to lymphatic hyperplasia”. Nonetheless, it is understandable that the number of resected lymph nodes corresponds to the extent of lymphadenectomy but, on the other hand, it cannot stand for the completeness of surgery as regional treatment.

## Existing Evidence

So far six randomized trials compared “limited” to “extended” lymphadenectomy as appreciated by their authors. The retrieved lymph nodes and other features of the trials are depicted in Table 1. Despite their major or minor limitations and the issues left unresolved, these trials contributed much not only to our knowledge regarding gastric cancer but also in general.

**Table 1:** Prospective randomized trials comparing D_1_ to D_2_ Lymphadenectomy.

First author, major publications		Country	Study period	Patients enrolled		Resected Lymph Nodes			
						Average		Median	
				D_1_	D_2_	D_1_	D_2_	D_1_	D_2_
1	Dent et al (12)	South Africa	1982–1986	22	21	NR	NR	NR	NR
2	Robertson et al (13)	Hong Kong	1987–1991	25	30	NR	NR	NR	NR
3	Bonenkamp et al (14–17)	Holland	1989–1993	380	331	17	30	NR	NR
4	Cuschieri et al (18, 19)	UK	1986–1993	200	200	NR	NR	13	17
5	Wu et al (20–22)	Taiwan	1993–1999	110	111	19	37	NR	NR
6	Degiuli et al (23, 24)	Italy	1998–2005	133	134	28	37	25	35

NR, not reported

- The trial of Dent et al (12) in South Africa randomized 43patients. Major findings were that blood transfusion requirements, operating time and hospital stay were longer with extended lymphadenectomy. At a median follow-up of 3.1 years no benefit regarding survival was seen.
- Robetson et al (13) randomized 55 patients in Hong Kong into D_1_ or D_2_ lymphadenectomy with compulsory pancreaticosplenectomy to find out again that operating time, transfusion requirements and hospital stay, all increased with extended lymphadenectomy. Morbidity was also higher with extended lymphadenectomy and was mostly due to abdominal sepsis. Contrary to the expectations overall survival was significantly worse and this was attributed to the impact of increased blood transfusion.
- Cuschieri et al (18, 19) in the UK randomized 400 patients into two equal arms. 8 patients of the D_1_ arm and 113 of the D_2_ arm had a pancreaticosplenectomy while 54 patients of the D_1_ arm and 18 patients of the D_2_ arm had a splenectomy. Pancreas and/or spleen resection were blamed for the significantly higher morbidity seen after extended lymphadenectomy and the significantly longer hospitalization. Hospital mortality was also significantly higher. The 5-year Overall Survival, Disease Specific Survival and Disease Free Survival were similar in both treatment arms. The authors speculated that the possible benefit of extended lymphadenectomy was lost by the detrimental effects of pancreatico-splenectomy. The longest survival of the trial was seen with pancreas and spleen preserving D_2_-lymphadenectomy. But then again this concerned patients with more distal tumors, which may have acted as a cofounding factor.
- In the “Dutch trial” (14–17) 380 patients underwent D_1_- and 331 D_2_- lymphadenectomy. 11% of the D_1_-patients and 38% of the D_2_- patients had a splenectomy. Among them 3% of the D_1_-patients and 30% of the D_2_-patients had a pancreatico-splenectomy. Again extended lymphadenectomy increased mortality, morbidity, reoperation rates and hospitalization. After 15 years the D_2_- group had a non-significant better Overall Survival, Disease Free Survival and Recurrence Risk. In contrast, Overall Recurrence Pattern and Gastric-Cancer-Related Death Rate were significantly lower. Patients with D_2_-lymphadenectomy without pancreatico-splenectomy showed again a significantly higher Overall Survival.
- In the Taiwanese trial (20–22) conducted by Wu et al , 211 patients were randomly allocated into D_1_- or D_2_-lymphadenectomy. Extended lymphadenectomy increased operating times, blood loss, transfusion and hospital stay. Again morbidity was increased mostly due to abdominal sepsis but mortality did not differ. Extended lymphadenectomy led to significantly higher 5-year Overall Survival but no difference in the Recurrence Rates was seen in the cases with R_0_ resection.
- Due to poor accrual, the Italian study (23, 24) closed after 8 years with a statistical power of approximately 80% as only 267 patients were randomized. Morbidity, mortality, 5-year Overall Survival and Disease Specific Survival were similar in both arms. However limited lymphadenectomy led to significantly better Disease Specific Survival in patients with pT_1_ tumors and patients older than 70-years old.

## Is D_2_ Lymphadenectomy More Effective Than D_1 _?

As it can be seen in Table 2 the Taiwanese trial (which excluded patients with early gastric cancer) demonstrated a benefit of D2-lymphadenectomy on 5-year Overall Survival and 5-year Disease Specific Survival (22). Similarly, the Dutch trial showed that D_2_ lymphadenectomy reduces significantly Reccurence Rates and Gastric Cancer Related Death after 15 years (17) In all other trials overall Survival Rates were similar after D_1_ and D_2_ dissection (12, 13, 17, 19, 22, 24). Regarding the oncological outcome of extended lymphadenectomy “stratified” by cancer stage, the univariate analysis of the Dutch trial data revealed a significant benefit for 15-year overall survival for Stage II and for N_2_ patients (by TNM 1997 definition) however the outcome of multivariate analysis was not reported (17). In contrast, the Italian trial showed that D1 lymphadenectomy may be better in aged patients and in early gastric cancer (pT_1_ disease D1: 98% vs D2: 83%, *p* = 0·015) (24). Nevertheless, none of the trials had enough power to reveal a survival difference for a certain cancer clinical TNM- or T-, or N- stage after extended lymphadenectomy. Also, the same outcome seemed to concern patients with early or with locally advanced cancer. None of the trials offered solid data for the election of the extend of lymphadenectomy in proportion with the disease burden. Of note, that none of the trials including Stage IA patients discriminated between T_1a_ and T_1b_ patients. This distinction seems of great importance as invasion of submucosa changes the malignant potential of the disease.

**Table 2:** Comparison of D_1_ to D_2_ Lymphadenectomy (D_1_:D_2_) in prospective randomized trials regarding oncologic outcome.

**First author & publication**	**Country**	**OS (%)**					**DFS %**	**DSS %**	**GCRD**
		3 Year	5 Year	11 Year	15 Year	RR %	5-year	5-year	15 Year
Dent (12)	South Africa	78 vs 76							
Robertson (13)	Hong Kong		48 vs 40						
Cuschieri (19)	UK		35 vs 33				41 vs 41	42 vs 43	
Wu (22)	Taiwan		60 vs 53*			51 vs 40		65 vs 58*	
Bonenkamp (15)Hartgrink (16) Songun (17)	Holland		47 vs 45	30 vs 35	22 vs 28	43 vs 37	44 vs 42	66 vs 58	48 vs 37*
Degiuli (24)	Italy		67 vs 64					71 vs 73	

* *p* < 0.05

OS, overall survival; DFS, disease free survival; DSS, disease specific survival; GCRD, gastric cancer related death.

A number of meta-analyses of the final results from these randomized trials have been published also. Their conclusions regarding survival are summarized in Table 3 but as seen in the I^2^ column most of them suffer from heterogeneity. Also the duration of follow-up differs among the included studies. Mocellin et al (25, 26) included the 15 years results of the Dutch trial and Jiang et al (28) included the 10-year Dutch results. The rest of the included studies in all meta-analyses were based on the 5-year results. Despite these shortcomings, these meta-analyses still worth consideration. Most of them concluded that no difference seems to exist between D_2_ and D_1_ lymphadenectomy regarding Overall Survival. Only a meta-analysis of the subgroup of Hong Kong and Taiwan trials combined led to a significant improvement of overall survival, but this was at the cost of extremely high and significant heterogenity (26). In contrast, the subgroup of the European studies was highly homogenous but showed no difference between the two arms (26). Similarly Disease Free Survival was unaffected, but D_2_-dissection improved significantly Disease Specific Survival (25, 26). Along the same vein Jiang et al found that D_2_ dissection decreased significantly the risk of Gastric Cancer Related Death when the British trial was excluded as a cause of heterogeneity (28). Finally, El Sedfry et al showed that D_2_-dissection improves 5-year Overall Survival of patients with pT_3_ disease, but not of those with early gastric cancer or pT_2_ disease, a finding opposing the Italian study (27). This evidence altogether is not compelling but cannot be ignored and favor extended lymphadenectomy

**Table 3:** Comparison of D_1_ to D_2_ Lymphadenectomy in meta-analyses regarding oncologic outcome.

	First author (reference)	Included trials	OS D_2_ vs D_1_	CI	*p*	I^2^	*p*(I^2^)
OS	Mocellin (25, 26)*	H, B, T, D, I	HR: 0.91	0.71–1.17	0.47	64%	**0.024**
	Mocellin (26)	H, T	**HR: 0.63**	0.40–0.99	**0.047**	76%	**0.039**
	Mocellin (26) *	B,D,I	HR: 0.98	0.86–1.12	0.8	0%	0.81
	El-Sedfy (27)	B, T, D, I	OR: 1.11	0.84–1.47	0.47	45%	0.14
	Jiang (28),*	H, B, T, D, I	RR: 0.98	0.88–1.09	0.74	0%	0.78
	El-Sedfy (27) 5 year pT1	B, T, D, I	OR: 0.60	0.23–1.57	0.29	58%	0.09
	El-Sedfy (27) 5 year pT2		OR: 1.05,	0.67–1.64	0.83	31%	0.24
	El-Sedfy (27) 5 year pT3		**OR: 1.64**	1.01–2.67	**0.05**	0%	0.94
	El-Sedfy (27) 5 year pN+		OR: 1.36,	0.98–1.87	0.06	0%	0.45
	El-Sedfy (27) 5yr N0		OR: 0.77	0.49–1.22	0.26	23%	0.26
DSS	Mocellin (25, 26)*	B, T, D, I	**HR: 0.81**	0.71–0.92	**0.002**	40%	0.17
DFS	Mocellin (26)*	B, T, D,	HR: 0.95	0.84–1.07	0.37	36%	0.21
GCRD	Jiang (28) *	B, T, D,	RR: 1,19	0.98–1.44	0.07	55%	0.11
	Jiang (28) *	T, D,	**RR: 1,31**	0.12–1.52	**<0.001**	0%	0.76

* Duration of follow up varies in the included studies.

OS, Overall survival; DFS, disease free survival; DSS, disease specific survival; GCRD, gastric cancer related death.

CI, 95% CI; HR, Hazard ratio; OR, Odd ratio; RR, relative risk; 5 year, 5 year follow up.

H, Hong Kong; B, British; T, Taiwanese; D, Dutch; I, Italian study.

## Is D_2_ Lymphadenectomy Safe? Morbidity and Mortality

The technical complexity and difficulty of D_2_ – lymphadenectomy rose concerns on its morbidity and mortality. Tables 4; 5 depict the outcomes of the comparison between D_1_ and D_2_ lymphadenectomy in randomized trials and subsequent meta-analyses. Both the British and the Dutch trial and two recent meta-analyses of their data (14, 18, 25, 28) revealed increased mortality after D_2_ dissection. In the rest trials there was no difference in mortality. All trials except the Italian found increased morbidity and hospitalization with D_2_-gastrectomy (12–14, 18, 20, 21). Significantly more time was needed for the D_2_- operation as shown by the South African, Hong Kong and Taiwan trials (12, 13, 20, 21). Also in the same trials and the Dutch one D_2_- lymphadenectomy led to significantly greater blood loss and transfusion requirements (12–14, 20, 21). This was refuted by a subsequent meta-analysis which on the other hand showed that D_2_- dissection increased significantly all other complications: anastomotic leaks, pancreatitis, pulmonary complications, wound infection rates and re-operation rates (28).

**Table 4:** Morbidity, mortality and perioperative characteristics in prospective randomized trials comparing D_1_ to D_2_ Lymphadenectomy.

First author, (reference)	Morbidity		Mortality		Op. Time (hrs)		mean LOS		Transfusion (units)		Transfused patients	
	D_1_	D_2_	D_1_	D_2_	D_1_	D_2_	D_1_	D_2_	D_1_	D_2_	-	-
Dent (12)	14%	36%	0%	0%	1,7	2,3*	9,3	13,9*	4	25*	-	-
Robertson (13)	0%	58%	0%	3%	2,3	4,3*	8	16*	0	2*	7	23*
Cuschieri (18)	28%	46%*	5%	11%*			18	23*	-	-	-	-
Wu (20, 21)	7%	17%*	0%	0%	3,6	4,5*	15	19,6**	1,3	2,1*^†^	-	-
Bonenkamp (14)	25%	43%*	4%	10%*	-	-	18	25*			113	170*
Degiuli (23)	12%	18%	3%	2%			13	13				

* *p* < 0.05

† blood loss

LOS, Length of hospital stay; Op. time, operative time.

**Table 5:** Morbidity and mortality in meta-analyses comparing D_1_ to D_2_ Lymphadenectomy.

D_1_ vs D_2_		CI	*p*	I^2^	*p*(I^2^)	First author(reference)	Included trials
Anastomotic Leakage	**RR: 0.47**	0.31–0.71	**<0.001**	0	0.42	Jiang (28)	S, H, B, T, D, I *^†^
Pancreatic Leakage	**RR: 0.47**	0.13–0.76	**0.01**	0	0.97		H, T, D, I *^†^
Reoperation Rate	**RR: 0.46**	0.28–0.76	**0.002**	7	0.37		H, T, D, I *^†^
Haemorrhage	RR: 0.69	0.36–1.33	0.27	5	0.38		H, T, D, I *^†^
Wound infection	**RR: 0.51**	0.32–0.83	**0.006**	0	0.53		S, B, T, D, *^†^
Pulmonary Complications	**RR: 0.48**	0.33–0.71	**<0.001**	0	0.58		S, B, D, I *^†^
							
Mortality D1 *vs* D2	**RR: 0.58**	0.47–0.71	**<0.001**	0%	0.68	Jiang (28)	S, H, B, T, D, I *^†^
Mortality D2 *vs *D1	**RR: 2.02**	1.34–3.04	**<0.001**	0%	0.66	Mocellin (25)	H, B, T, D, I

* Dutch Data from Hartring et al 2004  (16)

† Includes data from Li Wei Wen et al 2007  (29)

CI, 95% CI; RR, relative risk.

S, South African; H, Hong Kong; B, British; T, Taiwanese; D, Dutch; I, Italian.

## The Role of Pancreas And/or Spleen Resection in Morbidity and Mortality

A major part for the increase of morbidity and mortality has been attributed to the resection of the spleen and/or the pancreas, once though a compulsory step for D_2_-lymphadenectomy (Tables 6, 7). Although the Italian trial showed that post-operative hospital stay was not affected by splenectomy implying unaltered morbidity (23), the Taiwanese and the UK trial reported an increase in morbidity after both splenectomy and/or pancreaticosplenectomy (18, 20, 21). Furthermore, the difference in morbidity between D_1_- and D_2_- lymphadenectomy became non-significant in the British trial after allowance for splenectomy or pancreatico-splenectomy (18). In other words the increased morbidity seen after D_2_ lymphadenectomy was chiefly due to the removal of pancreas and/or spleen. In contrast, the Dutch trial reported no association of the increased morbidity of D_2_- lymphadenectomy to pancreaticosplenectomy (14). With regards to mortality both the Dutch and the Taiwanese trial reported no effect of pancreatico-splenectomy (14, 20, 21). In contrast, the British trial reported a significant increase of mortality after pancreatectomy and/or splenectomy (18). This was confirmed by a meta-analysis of the British and Dutch data which also showed that postoperative mortalities of D_1_- and D_2_- dissection were even after resection of the spleen and/or the pancreas (Table 8) (28) .

**Table 6:** Patients with Splenectomy or Pancreaticosplenectomy in the prospective randomized trials comparing D_1_- to D_2_-Lymphadenectomy.

	**Country**	**Sample size** D_1_:D_2_	**Splenectomy** D_1_:D_2_	**Pancreatectomy** D_1_:D_2_
1	South Africa	22:21	0:0	0:1
2	Hong Kong	25:29	0:29	0:29
3	UK (MRC)	200:200	62:131	8:113
4	Taiwan	110:111	3:1	1:13
5	Holland	380:331	41:124	10:98
6	Italy	133:134	9:12	2:2

**Table 7:** Effect of Splenectomy and Pancreatico-splenectomy on morbidity, mortality and oncologic outcome in prospective randomized trials comparing D_1_- to D_2_-Lymphadenectomy.

	**Country**	**D_1_/D_1Sple_**	**D_2_/D_2Spl_e**	**D_1_/D_1Pancr_**	**D_2_/D_2Pancr_**	**D_1_/D_2_ -**	-**^/^pancr**	-**^/^sple**
Mean OS	Holland	7.37/5.14*	9.09/5.19*	7.27/2.34*	8.81/4.85*			
15 year OS % (17)	Holland					22/35*		
5 year OS% (22)	Taiwan					54/61*		
5 year DSS% (22)	Taiwan					57/64*		
5 year OS% (19)	UK	35/39	46/33	35/13	46/25	35/46	39/24/38 pancr/sple*	
Morbidity % (20, 21)	Taiwan						10.6/35,7*	
Morbidity% (18)	UK	20/44	22/59	28/-	30/58		28/56*	28/54*
Mortality % (18)	UK	4/13	6/17	6/-	9/16		7/16*	9/16*

D_1_/D_1Sple_: D_1 _*vs* D_1_-dissection with splenectomy, D_2_/D_2Sple_ : D_2_ vs D_2_-dissection with splenectomy,

D_1_/D_1Pancr_: D_1_*vs *D_1_-dissection with pancreaticosplenectomy, D_2_/D_2Pancr_: D_2_ vs D_2_-dissection with pancreaticosplenectomy,

D_1_/D_2_ - : D_1_*vs *D_2_ both without pancreaticosplenectomy,

pancr: All patients without vs all patients with pancreaticosplenectomy , -/sple: All patients without vs all patients with splenectomy.

* p < 0.05

OS, overall survival; DSS, Disease Specific Survival.

**Table 8:** Effect of Splenectomy and Panceatico-splenectomy in mortality and oncologic outcome in meta-analyses comparing D_1_- to D_2_-Lymphadenectomy.

	REF	Included studies		95% CI	*p*	I^2^	*p*(I^2^)
D_1_*vs* D_2_ OS without Pancreaticosplenectomy	(30)	B, T, D^‡^	**HR = 0. 65**	0.52–0.80	**<0.0001**	19%;	0.29
D_1_*vs *D_2_ Mortality with Pancreaticosplenectomy	(28)	B, T, D *^†^	RR: 1.35	0.45–4.05	0.6	0%	0.48
D_1_*vs *D_2_ Mortality with Splenectomy	(28)	B, D *^†^	RR: 0.85	0.47–1.54	0.6	0%	0.66
D_2_ Mortality with *vs *without pancreaticosplenectomy	(28)	B, D *^†^	**RR: 0.46**	0.26–0.81	**0.008**	0%	0.39
D_2_ Mortality with *vs *without Splenectomy	(28)	B, D *^†^	**RR: 0.28**	0.14–0.56	**0.001**	0%	0.62

* Plus the study of Li  (29)

† Dutch data from Bonenkamp et al 1999  (15)

‡ Dutch data from Songun et al 2010 (17)

OS, Overall Survival; CI, 95% CI; HR, Hazard ratio; RR, Relative risk.

B, British; T, Taiwanese; D, Dutch; REF, reference.

Taken all these together we may conclude that D_2_-dissection leads to increased morbidity which on the other hand can be decreased significantly by avoiding resection of the spleen and the pancreas unless properly indicated.

## The Role of Pancreas And/or Spleen Resection on Survival 

Pancreatico-splenectomy, but not splenectomy alone, decreased Overall Survival significantly in the UK trial (19)(Tables 7 and 8). After 5 years the group with D_2_-dissection plus pancreaticosplenectomy presented the poorest survival. It was speculated that the possible benefits of extended lymphadenectomy were thus blurred. In the Dutch trial splenectomy or pancreaticosplenectomy decreased mean Overall Survival after 15 years in both the D_1_ and D_2_ dissection group (17). Interestingly, D_2_ lymphadenectomy without pancreaticosplenectomy led to a significant improvement of 15-year Overall Survival. This was supported by the Taiwanese trial where extended lymphadenectomy with preservation of the pancreas and spleen increased 5 year Overall Survival and Disease Free Survival (22). Of note, neither pancreatectomy nor splenectomy seemed to affect the Hazard Ratio for death (22). A meta-analysis of the data from the Dutch, the British and the Taiwanese trials regarding patients with and without splenectomy and/or pancreatectomy showed a benefit of D_2_- compared to D_1_-lymphadenectomy on Overall Survival. Remarkably the heterogeneity among the included trials was low (30). Given the known effect of pancreaticosplenectomy on morbidity, its detrimental effect on survival is not unexpected. Long term survival after oncologic gastrectomy is known to be influenced by morbidity and blood transfusion rate (31–35). As a consequence avoiding spleen and/or pancreas resection increases survival, at least indirectly.

## Protocol Violations and Other Issues

A strict adherence to the study protocol is of paramount importance in any clinical trial. Quality control in the comparison of two lymphadenectomy strategies should ensure the complete removal of the lymph node stations as predetermined by the protocol, and only these. Removing more or less stations constitutes protocol violation. If fewer stations are removed then compliance to the protocol is defective, if more stations are resected then contamination ensues. In particular for lymphadenectomy in gastric cancer, contamination has been defined as the resection of more than two lymph node stations that should not have been removed i.e., over-resection (36). On the other hand, non-compliance has been defined as the non-resection of more than two lymph node stations that should have been excised i.e., under-resection (36). Contamination of D_1_-operations and non-compliance of D_2_-operations would obviously mitigate any expected difference. As it can be seen in Table 9, in all European Trials more than 50% of the enrolled patients received something between D_1_- and D_2_- dissection regardless of the allocated treatment. Proving a difference between the two lymphadenectomies is difficult under these circumstances. This can be understood by the re-analysis of 15-year data from the Dutch trial. The overall survival initially reported had a difference of 8% (21% *vs *29% *p* = 0.351 for D1 and D_2_-dissection respectively). After excluding both non-compliant and contaminated operations the difference became 10% (23% *vs *33% *p* = 0, 26 for the D_1_ and D_2_ groups respectively) but still remained not significant (37). However the 15-year survival of the group of 139 patients with a compliant and contaminated D_2_ dissection (i.e., those who had a proper D_2_-resection and beyond) was higher than the survival of the non-contaminated D_1_-group (those who had a proper D_1_-dissection); and more importantly it was higher than the survival of the remaining 192 patients who underwent what the authors perceived as “D_2_ dissection” (37).

**Table 9:** Protocol violations confusing the comparison of D1 vs D2-Lymphadenectomy.

	**Country of Study**	**Contaminated D_1_**	**Non-compliant D_2_**	**Protocol Violation (D_1½_ ?)**
1	South Africa	-	-	
2	Hong Kong	-	-	
3	UK	6	52	58
4	Taiwan	1	0	1
5	Holland	6	51	57
6	Italy	18	34	52

The role of training and of the learning curve also cannot be overemphasized. Conducting a proper D_1_- or D_2_- lymph node dissection and being able to discriminate between these two operations demands appropriate training. This improves operative time, lymph node yield, morbidity and mortality. Parkih et al in UK reported that the curve reaches a plateau after 18—24 months or 15–25 procedures (38), Lee et al reported that 8 months or 23–35 cases are needed (39) while Wu et al defined the number of needed cases to 25 (21). Furthermore training influences oncologic outcome because Overall Survival is significantly improved by a history of 100 gastrectomies (40). Such an in-depth training was reported to exist for the surgeons of the Taiwanese trial. Also in the Italian trial all surgeons had a history of at least 21 D_2_ dissections due to their participation in the Italian multicenter phase II study (41). Consequently, they were adequately trained regarding morbidity and mortality. As expected, morbidity and mortality in the Taiwanese and Italian studies were more or less the same. In contrast in the Dutch trial the training actually happened during the study. As the investigators state, during the first 4 (42) or 6 months (14) one Japanese surgeon trained the 8 (42) or 11 (14) regional supervising surgeons (the training period and number of regional supervisors-trainees are unclear in literature). They in turn, supervised and advised the regional surgeons. Additional training material (booklet and videotape) were given to the regional surgeons. Also during the trial the Japanese and/or a supervising surgeon proctored the regional surgeons (14, 42). 67 patients were allocated to the D_2_-arm in the first 10 months of the study i.e., the training period and some more months. The Japanese instructor operated 34 patients and the supervising surgeons 33 (42). In other words, by the time each supervisor started supervising and proctoring the regional surgeons he himself had performed the maximum of 4 D_2_ dissections (42). The trial went on and, surprisingly, no learning curve was detected for any surgeon. Similarly, in the British trial the operating surgeons were trained with a booklet and an instructional video. Standardization was also pursued through an “operative form” filled by the surgeon. No proctorship or mentorship was available throughout the trial (18). Given the comparable training of surgeons in these two European trials the similarity in the morbidity and mortality outcomes is not unpredictable. Regarding the South African and the Hong Kong trial, no data exist on the training of the participating surgeons and no comments can be made. Finally, as far as the oncologic outcome is concerned the criteria defined by Kim et al (40) are fulfilled only by the Taiwanese trial, which of note is the only one who demonstrated a survival difference.

Beyond any doubt the comparison of two different surgical strategies against a known lethal disease such as gastric cancer should have survival as a chief endpoint. However Overall Survival includes also all those patients who died from causes unrelated to gastric cancer. Although theoretically these patients will be equally distributed in the arms of a well conducted randomized comparison, this is not always the case. For example, the patients of the D_2_ arm were accidentally older in the South African trial (12), and there is always a possibility of unknown cofounding factors not taken into account. Disease Specific Survival and Disease Related Death Rate are more specific endpoints because they reflect directly those who survived and those who died from gastric cancer. The validity of these endpoints can be seen in the Dutch trial where no difference was seen in Overall Survival but the difference in Gastric Cancer Related Deaths i.e., the principal aim of surgery was statistically significant (17).

Any trial should have the power to prove the expected difference in the treatment outcome. One can speculate that this was not probably the case in the trials of Dent et al (12) and Robertson et al (13) where the size of each arm was small. Furthermore, the population where the trial is conducted should be of enough size to support the accomplishment of the trial. This was not the case with the Italian study which closed due to pure accrual (24). Moreover, the expected outcome study should be clearly and pragmatically defined. This can be seen in the British trial where the initial estimate was a 20% survival for the D_1_ arm and a 12% improvement with D_2_ dissection (19). These estimates were incorrect and survival after D_1_-dissection was found to be around 30% (19, 43). To prove a 10% improvement in the outcome more than 1,000 patients should have been enrolled and the recruitment period would have exceeded 15 years (43), which brings us to the next issue: the timeframe during which a trial is conducted must be narrow. During the years medical care improves, patients are diagnosed earlier, staging becomes more accurate, perioperative care becomes more effective, postoperative treatment evolves; even surgery itself changes with the implementation of endoscopic, minimally invasive and other techniques.

## Conclusion

From the studies conducted so far there is plenty of evidence on the equivalence, and there is limited evidence on the superiority of D_2_- lymphadenectomy. If morbidity and mortality are kept to a minimum, then probably D_2_ lymphadenectomy is advantageous, in particular for patients with locally advanced tumors. However it seems that this advantage is easily wiped out by unrequired resection of the pancreas and/or spleen and by inadequately trained surgeons. It has to be noted however that the existing studies are neither recent nor flawless and the conclusions drawn may not pertain to today’s modern medicine. Since the closure of the last trial on the row, some 12 years ago, much progress has been made in the diagnosis, staging, peri-, and post-operative care of patients with gastric cancer. Perhaps in the near future as medicine advances, the necessity and extend of lymphadenectomy will have to be redefined. The new framework of surgical oncology should be individualized and take into consideration “novel” parameters like neoadjuvant treatment, biological therapies and, more importantly, the molecular characteristics of each tumor. Until new solid evidence appears, with new, contemporary, well designed and properly conducted randomized trials, implementation of D_2_-lymphadenectomy into the daily practice of well-trained surgeons seems justified. For this reason, the vast majority of the scientific societies worldwide issued guidelines with D_2_- lymphadenectomy as the standard treatment for resectable gastric cancer (44–49) provided that surgical expertise and postoperative care are of sufficient standard and that treatment will be delivered in high volume specialized centers.

## Author Contributions

IK and AM both participated on the conception and design of the manuscript; data acquisition, interpretation and analysis; drafting, revising and final approval of the manuscript.

## Conflict of Interest Statement

The authors declare that the research was conducted in the absence of any commercial or financial relationships that could be construed as a potential conflict of interest
